# How is trauma-focused therapy experienced by adults with PTSD? A systematic review of qualitative studies

**DOI:** 10.1186/s40359-024-01588-x

**Published:** 2024-03-09

**Authors:** Solveig Flem Gjerstad, Linda Nordin, Stig Poulsen, Erminio Francesco Antares Spadaro, Sabina Palic

**Affiliations:** 1 Department of Psychology, University of Copenhagen, 2A Oester Farimagsgade, 1353 Copenhagen, Denmark; 2 DIGNITY- Danish Institute Against Torture, Copenhagen, Denmark; 3 Department of Psychology, Lund University, Lund, Sweden; 4 Department for Treatment of Borderline Personality Disorder and Self-Harm, Psychiatric Centre Glostrup, Mental Health Services in the Capitol Region of Denmark, Copenhagen, Denmark; 5 Clinical Department of Eating Disorders, Mental Health Centre Ballerup, Mental Health Services in the Capitol Region of Denmark, Copenhagen, Denmark

**Keywords:** Trauma-focused therapy, Systematic review, Qualitative research, Patient experience

## Abstract

**Background:**

Trauma-focused therapies (TFTs) are first-line treatments for posttraumatic stress disorder (PTSD). However, TFTs are under-utilised, partly due to clinicians’ and patients' fear that TFT is too challenging or harmful. We review the qualitative studies on how adults with PTSD experience TFTs to enhance the understanding of user perspectives, therapeutic processes, and outcomes.

**Methods:**

PubMed, PsychINFO and PTSDPubs were searched between October 1st and November 30th, 2021. Study quality assessments were undertaken, and studies were analysed using a descriptive-interpretative approach. Nine studies were included.

**Results:**

The analysis resulted in the identification of four key domains, representing a temporal sequence of TFT stages: Overcoming ambivalence towards TFT, Experience of treatment elements, Motivation for dropout/retention, and Perceived changes post-treatment.

**Conclusion:**

Although many participants reported high levels of distress and considered dropping out, only a minority did eventually drop out and most patients expressed that the hardships in therapy were necessary for PTSD improvement. Establishing a safe therapeutic environment and working with the ambivalence towards treatment was essential for retention. This review serves a dual purpose, to shed light on diverse TFT experiences found to be important for treatment satisfaction, and to elucidate common treatment patterns. The results can be used in preparing patients for therapy and in training TFT therapists. Studies had moderate to high quality, and more studies of experiences of TFT non-responders and dropouts in a non-veteran population are needed to further our understanding of the utility and limitations of TFTs.

**Supplementary Information:**

The online version contains supplementary material available at 10.1186/s40359-024-01588-x.

Posttraumatic Stress Disorder (PTSD) is a highly prevalent psychological disorder characterised by symptoms of re-experiencing trauma-related memories, avoidance, and elevated arousal [1]. The disorder is more commonly reported in high-income countries; however, the symptoms, personal and socioeconomic consequences are consistent globally [2]. Several types of therapies have been developed with various degrees of proven efficacy. Trauma-focused therapies (TFTs) are suggested as the first-line treatment for PTSD [3]. In a summary of the major Clinical Practice Guidelines (CPGs), Hamblen et al. [4] found that all guidelines [5–9] strongly recommended trauma-focused cognitive behavioural therapy (TF-CBT), including cognitive processing therapy (CPT) [10] and prolonged exposure (PE) [11]. Eye movement desensitisation and reprocessing (EMDR) [12] was strongly recommended by all except one guideline [5].

For this review, TFT is defined as “any therapy that uses cognitive, emotional or behavioural techniques to facilitate the processing of a traumatic experience and in which the trauma focus is a central component of the therapeutic process” [13]. Specific types of TFT include, but are not limited to, cognitive-behavioural therapies such as PE, CPT, cognitive therapy for PTSD (CT-PTSD) [14], and narrative exposure therapy (NET) [15], as well as EMDR, an alternative format of delivering exposure therapy in association with cognitive and emotional components [13]. In the literature, TF-CBT has both been described as a category encompassing the various cognitive-behavioural therapies mentioned above, or as a specific type of TFT [16]. In this review, TF-CBT will be referred to as an overarching category of cognitive-behavioural treatments, and TFT will be used as a broader category including TF-CBTs and EMDR.

The distinctive TFTs utilise different interventions. However, they are thought to create change in the same underlying mechanisms, targeting emotion, cognitions, and avoidance of trauma memories [17]. The cognitive model of PTSD [18] explains exposure, i.e., imaginal or written reliving and in-vivo exposure, as essential because it modifies negative trauma appraisals. The emotional processing theory [19] further posits that inhibition of the exaggerated fear response occurs when the patient experiences an optimal emotional activation while experiencing information contradicting their fears. Thus, TFT is theorised and expected to be challenging, as it requires high levels of activation of negative emotions during exposure.

Despite TFTs being the recommended first-line treatments for PTSD, they are often not utilised in clinical practice [20]. A review by Finch et al. [21] found that three of the most important clinician-related barriers to the use of the evidence-informed interventions are fear of re-traumatising patients, fear of increasing patients’ symptoms, and a disliking of the inflexibility of the manualized approaches. Therapists further often believed TFT to be unsuitable for patients in case of comorbidity or more complex trauma histories [21]. However, evidence does not support the concerns regarding the retraumatization and un-suitability of TFTs in cases with comorbidity, for example for psychosis [22], dissociative symptoms [23] and major depressive disorder [24]; see also Ennis et al. [25] and Voorendonk et al. [26].

Smith et al. [27] reviewed barriers to help-seeking in adults with PTSD, including barriers to initiating TFT. The findings revealed complex patterns, where some participants reported not being emotionally ready to talk about trauma or believing PE or CPT to be ineffective or harmful [28–30], and others having a strong preference for exposure therapy [31]. Importantly, the patients’ treatment preferences impacted therapists’ willingness to offer TFT [21]. Hence, systematic reviews [21, 27] find that fear of negative experiences and reactions to TFT in both patients and therapists alike is a central barrier to its application.

Finally, TFT has been associated with higher drop-out rates than non-TFT [32, 33], and the non-response rate to TFTs is often reported around 50% percent. This has been unchanged over the last two decades [33, 34] despite extensive quantitative research into predictors and moderators of outcome as well as therapy effectiveness and dismantling studies. Therefore, a qualitative review of TFT users’ experiences may inform us on how to more efficiently approach the TFT concerns of therapists and patients, as well as the questions of drop-out and nonresponse. Further, users’ experiences with TFTs may lead to new research questions and designs, which can not be deduced from quantitative associations between variables.

Hence, investigating similarities and discrepancies in *how* patients experience the TFTs might be essential in understanding how to close the gap between current clinical practice and treatment guidelines for PTSD. While reviews studying this question have been conducted for populations of children and youths [35] and patients’ experience with EMDR [36], as of today, no review has investigated adult patient experiences with TFTs.

## Aims

This qualitative review aims to synthesise adult patients’ reported experiences of TFTs to broaden the understanding of common and relevant treatment experiences, themes and trajectories in the therapeutic processes that can inform ways to more efficient and successful delivery of TFT.

## Methods

### Eligibility criteria

The study inclusion and exclusion criteria for this analysis were defined a priori, using the SPIDER mnemonic: Sample, Phenomenon of Interest, Design, Evaluation and Research type [37]. The final inclusion criteria were: English-language, published, peer-reviewed, qualitative and mixed-method studies reporting on the experience of receiving TFT for patients aged 17 or above with a primary diagnosis of PTSD. The inclusion age was modified from 18 to 17 years during the screening process since one identified article [38*] with participants aged 17– 25 years (M = 20.0, SD = 2.61) was assessed to provide important qualitative information. Since the mean age was 20 and only a few participants were below the age of 18, it was decided that the study should be included. Studies describing both positive and negative experiences of receiving TFT were included to ensure variation in the phenomenon and strengthen the fidelity to the subject matter [39]. Some studies were excluded from the analysis because they had been discussed in a prior systematic review, exploring patients’ experiences with EMDR [36]. Their findings are included in the discussion of the current review. However, as the review by Whitehouse [36] only included studies with clients identified as potentially benefiting from EMDR, older studies that did not meet the inclusion criteria for the review by Whitehouse [36] were considered for the current review. Therefore, studies involving EMDR published before Whitehouse’s review were also included.

### Search strategy

The systematic search consisted of three phases. The first phase involved a preliminary search in PubMed and Google Scholar to identify keywords within the abstract of relevant articles. The SPIDER [37] tool was applied to define search terms from the review question and develop a standardised search strategy. The second phase consisted of systematic searches using the identified keywords. The searches were conducted in three databases: Pubmed (Medline), PsycINFO (EBSCOHost) and PTSDPubs (Proquest) between October 1st and November 30th, 2021. Some of the search terms were modified to fit the different databases (e.g., using truncation and wildcards). The final phase consisted of hand searches in the chosen studies’ reference lists. The key search terms are presented in Table 1. For a comprehensive presentation of the search process for each database, see Additional file 1.

**Table 1:** Search terms used in PsycInfo

SPIDER	Search terms
Sample	“posttraumatic stress disorder*” OR “post-traumatic stress disorder*” OR “post traumatic stress disorder*” OR PTSD
Phenomena of interest (P of I)	“cognitive proces* therap*” OR “prolonged exposure*” OR TF-CBT OR "eye movement desensitization and reprocessing" OR “narrative exposure therapy” OR “brief eclectic psychotherapy” OR “written narrative exposure” OR (“trauma-focused” AND (“therapy” OR “psychotherapy” OR “cognitive behavio#r* therap*” OR “cognitive therap*” OR “behavio#r* therap*” OR “behavio#r psychotherapy*” OR “cognit* therap*” OR “cognit* psychotherap*”))
Design	questionnaire* or survey* or interview* or focus group* or “case stud*” or observ* or qualitative* or “thematic analy*” or content analy* or ethnog* or phenomenol* or emic or etic or hermeneutic* or “heuristic*” or semiotics or “field study*” or “lived experience*” or “narrative analy*” or “grounded theor*” or “multi-method*” or “mixed-method*” or triangula* or “formative evalua*” or “process evalua*”
Evaluation (E)	«self-report*» OR «patient* report*» OR «client* report*» OR «experience*» OR view* OR perspective* OR perce* OR opinion* OR understand* OR reflect* OR reaction* OR thought* OR standpoint* OR “patient* receptivit*” OR “client* receptivit*” OR satisfaction* OR “client record*” OR “patient* record*” OR attitude* OR feel* OR belie* OR know* OR thought* OR standpoint*
Research Type (R)	qualitati* or “mixed-meth*” or “mixed meth*” or “multi-meth*” or “multi meth*”
	S AND P of I AND (D OR E) AND R

This table presents the search terms used in PsycINFO. The same search terms were used in PubMed and PTSDPubs, with some alterations regarding truncation and wildcards to fit the databases. Limits used in the databases were: Publication Type (Peer Reviewed Journal); Language (English); Age Groups: (18 years & older). Adapted from “Users’ experiences of trauma‐focused cognitive behavioural therapy for children and adolescents: a systematic review and metasynthesis of qualitative research” by L. Neelakantan, 2019, *European Child and Adolescent Psychiatry, p.* 879

### Study selection

Studies were screened and reviewed by the first author with the aid of Covidence. First, duplicates were removed. Titles and abstracts were then screened for inclusion. Studies were retrieved and full-text assessed for eligibility. To limit the possibility of excluding relevant articles, credibility checks were conducted by auditing [40], meaning that the inclusion of some articles was discussed by three of the authors.

### Quality assessment

The quality of the included studies was ensured by including published, peer-reviewed studies [39]. Further quality assessment was undertaken using the Critical Appraisal Skills Programme [41], the most used quality checklist in health-related qualitative synthesis [42]. CASP does not provide a scoring system but allows for a systematic quality assessment through 10 questions with elaborative prompts. To summarise the quality assessment, questions 1 through 9 were evaluated with the following score: Yes/clearly described = 1; Partially described = 0.5; No/insufficient information = 0 (adapted from Neelakantan et al. [35]). The total scores were categorised as low quality (scores 0 – 3), moderate quality (scores 4 – 8) and high quality (scores 8—9). Studies identified as lower quality by the checklist were not excluded. The quality assessments were used to assess potential biases, reliability, and value in the review’s findings [43].

### Data analysis and synthesis

Descriptive-interpretative qualitative analysis [40] was applied to provide a comprehensive description of patients’ experience of receiving TFT, including ambiguities and differences found in the primary studies. This approach is suggested for qualitative meta-analyses because it minimises two opposite types of risks that qualitative studies face. One risk is being too relativistic but with insightful findings, meaning that the findings are relative to the interpreter of the analysis and cannot lead to a generalizable framework. Another risk is that the studies are too realistic but with superficial results, because the results simply reproduce the participants’ words true to their original form but lack essential comparisons and interpretation of importance across different studies [44, 45]. A qualitative systematic review not only provides a synthesis but also an interpretation of experiences, enabling the presentation of shared, divergent, or significant themes across various modalities of Trauma-Focused Therapies (TFT), group or individual settings, and spanning different types of trauma.

The analysis consisted of four steps, described by Timulak [40]. First, the collected data were assigned into domains, informed and adjusted by the data of the primary studies. The identified domains created a conceptual framework and represented a temporal sequence of participants’ experiences of trauma-focused therapy. Second, meaning units (the smallest units of the data that conveyed a clear meaning) were identified. Third, meaning units were clustered based on similarities, generating categories and sub-categories. Lastly, the main findings were abstracted in narratives, exemplified through direct quotes.

The analysis was guided by a realist epistemology, assuming a unidirectional relationship between patients’ reports and experiences [46]. Categories were identified at a semantic level, that is the explicit meaning of the data was identified and organised into patterns [46]. The patterns were interpreted in terms of their significance and broader meaning. The interpretation included assessing how the methodology in the primary studies may have influenced the results.

All contextual information, including direct quotes, descriptions, and discussions, were considered data. The rationale for including contextual information was to retain the meaning of the results and minimise the risk of overlooking the context of the primary studies, to which qualitative research is sensitive [40]. Only accounts available in the published versions were included. In mixed methods studies, only qualitative information was used as data. To minimise the risk of overgeneralizing findings, means were taken to address the representativeness of the results in the final review by reporting: (i) how many primary studies were included in each domain, category, sub-category and (ii) how many categories were identified in each study, to account for the degree that each study was represented in the final review [47].

The analysis and synthesis were initially conducted by the primary author. The fourth author then conducted a separate analysis and synthesis. The process of reaching consensus included making sure that the synthesis represented both analyses and that the final synthesis was adjusted to fit the categories of the independent analysis. Consensus of the domains, categories, meaning units and extracted data were reached in collaboration [48].

## Results

### Study selection

**Fig. 1:**
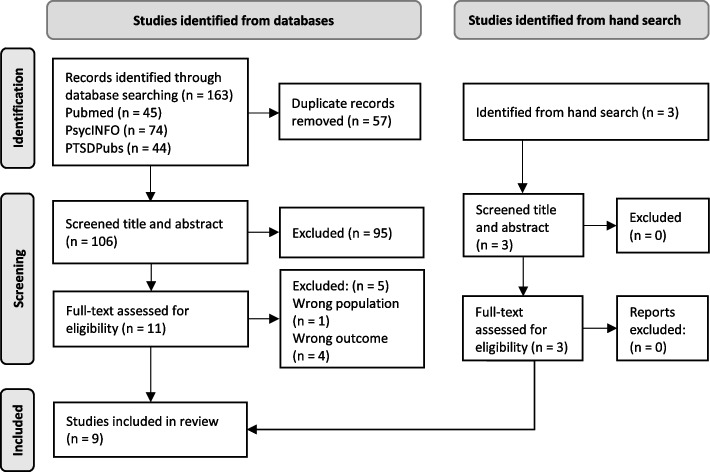
PRISMA flow diagram. Note: Articles excluded based on full-text assessment: Hundt et al. [28], König et al. [49], Sherril et al. [50], Tong et al. [51] and Wise & Marich [52]. From: Page, M. J. et al. (2021). The PRISMA 2020 statement: an updated guideline for reporting systematic reviews. BMJ, 372*,* n71. https://doi.org/10.1136/bmj.n71

The database search identified 163 studies, where 57 duplicates were removed. The resulting 106 records were screened based on title and abstract. After excluding 95 studies, 11 studies were retrieved and assessed for eligibility. Five studies were excluded during full-text screening, resulting in six studies identified from database-search. Three additional studies were identified through hand searches. All three studies were kept during the screening of title and abstract and the full-text eligibility assessment. The process resulted in nine included studies, three identified through hand-search and six from database searches. Details of the screening process are provided in a PRISMA Flow Diagram (Fig. 1).

The reasons for exclusion were wrong population (*n* = 1) and wrong objective (*n* = 4), i.e., not meeting the criteria for PTSD [51], investigating pre-treatment criteria [28], comparing the importance of treatment elements in two treatments without describing the experience [49], and not including qualitative information about how the patients’ experienced the treatment [50, 52]. For a more detailed description of the in- and exclusion process, see Additional file 2.

### Study characteristics

Table 2 provides details of study characteristics. The service contexts included trauma outpatient treatment services, a centre for anxiety disorders, veteran PTSD clinics, a youth mental health service and a primary care service. The studies described using TF-CBT, PE, CPT, Imaginary Rescripting (ImRs), EMDR, reliving, on-site-visits, Imaginal Reliving and/or Adapted Testimony within a TF-CBT framework. Two studies included group CPT, while the rest were delivered as individual therapy. Eight studies used qualitative interviews and one study used a questionnaire with free-text items. All samples consisted of adults, with one study focusing on young adults between 17 and 25 years. Reported traumatic events varied: physical and sexual assault or abuse, gang rape, road traffic accidents, medical trauma, military trauma, witnessing a murder, witnessing others or family members being killed, harmed or captured, finding a child murdered, physical torture, imprisonment, physical threat, domestic and childhood abuse, acts of terrorism, assaults with a weapon. The nine studies comprised 174 participants, with 38 dropping out of treatment (21.8%). Three studies reported interviews with dropouts. The rest of the studies recruited participants that had completed therapy.

**Table 2:** Study characteristics of primary studies

Study	Objective	Study Details			Study sample			Ethnicity or country of origin (n)	Trauma type (n)	Data Collection (Analysis)
		Intervention	Country	Dropout	Size	Age	Gender (male)			
Shearing et al. [53*]	Explore participants experience of reliving as part of TF-CBT	PE & CPT	UK	NR	N = 7	Range 20–50	14.3%	British (6), afro-Caribbean British (3)	Physical and sexual assault; road traffic accidents; other (NS)	Interview using topic guide (IPA)
Vincent et al. [54*]	Explore asylum-seekers experience of TF-CBT and its acceptability	Imaginal reliving and/or Adapted testimony	UK	NR	N = 7	M = 29 (SD = 7.5)	14.3%	Sudan (2), Zimbabwe (1), Afghanistan (1), Burundi (2), Iraq (1)	Physical assault (2), threats (1) and torture (1); sexual assault (3) witnessed others or family members harmed, killed, or captured (4), raped (2), torture (1), war (1), found child murdered (1), imprisonment (1)	Semi-structured interview (IPA)
Lowe and Murray [55*]	Understand aspects of TF-CBT that service users perceive as most useful	TF-CBT	UK	NR	N = 9	M = 53 (SD = 10.6)	44%	NS	NS	Semi-structured interview (IPA)
Murray et al. [56*]	Explore how site-visits, as part of TF-CBT, are experienced	TF-CBT	UK	NR	N = 25	M = 40.76 (Range 28–65)	73.9%	White British (15), Black British (4) South Asian (3), Middle-Eastern (1), white background (2)	Physical assaults (8), accidents (5), medical traumas (5), military trauma (2), witnessing a murder (2), domestic abuse (1), rape (1) and acts of terrorism (1)	Questionnaire with free text items (GT)
Hundt et al. [57*]	Understand veterans’ experiences of PE & CPT	PE & CPT	USA	NR	N = 23	M = 53.74 (SD = 12.2)	73.9%	Non-Hispanic white (8); African American (10); Native American (1); Asian (2)	NS	Interview using guide (GT)
Hundt et al. [58*]	Examine veterans’ experiences & reasons for dropping out of PE or CPT	PE & CPT	USA	100%	N = 28	M = 45.32 (SD = 15.2)	60.7%	Non-Hispanic white (7); African American (17); Hispanic Latino (4)	Combat (8); military sexual trauma (7); other military trauma (8); non-military trauma (5)	Qualitative interview (GT)
Boterhoven de Haan et al. [59*]	Explore patients’ and therapists’ experiences with TFT in patients with childhood trauma	ImRe or EMDR	Australia, Germany & Netherlands	NR	N = 13	M = 40 (SD = 12.2)	27.3%	Australian (12), German (25), Dutch (7)	Childhood trauma: sexual abuse/assault (23); physical abuse (13); other (8)	Semi-structured interview (TA)
Doran et al. [60*]	Understand veterans’ experiences with receiving PE and CPT in VA	CPT & PE	UK	44%	N = 18	M = 47.11 (SD = 15.3)	77.8%	Caucasian (10); African American (5); Hispanic/Latino (3)	Military trauma; non-military, sexual trauma; acts of war/perpetration; non-military, childhood abuse; other (NS)	Structured interview w. open-ended question (QCR)
Eastwood et al. [38*]	Explore young people’s experience of TF-CBT	TF-CBT	Australia	26%	N = 13	M = 20.0 (SD = 2.6)	30.8%	Australian (10); Aboriginal or Torres Strait Islander (1); Outside of Australia (3)	Physical assault (11); sexual assault (10); uncomfortable sexual experience (9); assault with a weapon (5)	Semi-structured interview (IPA)

*TF-CBT* Trauma-focused cognitive behavioural therapy, *PE* Prolonged exposure, *CPT* Cognitive processing therapy, *ImRs* Imaginary rescripting, *EMDR* Eye movement and desensitization reprocessing, *VA* Veteran Affairs Healthcare System, *GT* Grounded theory, *CQR* Consensual Qualitative Research, *TA* Thematic Analysis, *SD* Standard deviation, *NS* Not stated, *NR* Not relevant. For dropout, some studies are marked as not relevant because participants were recruited if they had completed therapy or trauma-focused intervention

### Synthesis of the results

**Fig. 2:**
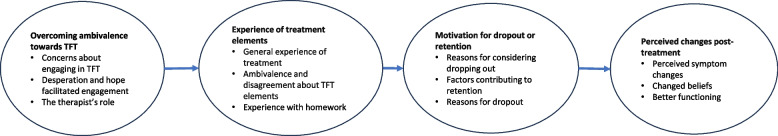
Experiences and dilemmas in different stages of TFT

The analysis resulted in a conceptual framework representing a temporal sequence of experiences and dilemmas in different stages of TFT. There were four key domains: *Overcoming ambivalence towards TFT*, *Experience of treatment elements*, *Motivation for dropout or retention* and *Perceived changes post-treatment*, with three categories for each domain. Each category was represented by several sub-categories (see Fig. 2 for a visual representation of experiences and dilemmas in different stages of TFT and Table 3 for a comprehensive summary of the distribution of the articles within each category and which studies are represented by the themes described below).

**Table 3:** Number of primary studies represented in each category

Domain, category and *sub-category*	Frequency	Studies represented
1. Overcoming ambivalence towards TFT	8/9	a, b, c, d, e, f, h, i
1.1 Concerns about engaging in TFT	5/9	a, b, d, f, h
*Fear of facing trauma*	4/9	a, b, d, h
*Scepticism about TFT’s effectiveness*	4/9	a, b, f, h
1.2 Desperation and hope facilitated engagement	5/9	a, b, c, e, g, i
*Social support*	2/9	a, c,
*Desperation*	4/9	a, b, c, e
*Determination and hope*	5/9	a, b, e, g, i
*Understanding and believing treatment rationale*	4/9	a, b, g, i
*Being in a good place in life*	1/9	a
1.3 The therapist’s role	6/9	a, b, c, d, f, h, i
*Collaborative process*	2/9	c, i
*Trusting the therapist*	4/9	a, b, d, h
*Pushed and respected*	5/9	a, b, c, d, i
*Empathy and authenticity*	5/9	a, b, c, f, i,
2. Experience of treatment elements	9/9	a, b, c, d, e, f, g, h, i
2.1 General experience of treatment	7/9	a, b, c, e, f, h, i
*Valued affective modulation strategies*	6/9	a, c, f, g, h, i
*Valued cognitive techniques*	5/9	c, e, f, h, i
*Psychoeducation created a sense of control*	5/9	a, b, c, e, i
*Length of therapy*	2/9	f, g
2.2 Ambivalence and disagreement about trauma-focused elements	9/9	a, b, c, d, e, f, g, h, i
*Harder than expected*	2/7	a, h
*Unfounded fears*	4/9	a, d, e, i
*Difficulty tolerating trauma work*	9/9	a, b, c, d, e, f, g, h, i
*Importance of hardship*	7/9	a, b, c, e, g, h, i
*Life consuming*	3/9	a, h, i
*Habituation*	5/9	c, d, e, h, i
*Piecing together traumatic experiences*	3/9	d, e, g
*Wanting less repetition*	1/9	h
2.3 Experience with homework	3/9	c, e, h
*Helped generalise learning*	2/9	c, e
*Difficult to complete*	2/9	e, h
3. Motivation for dropout or retention	7/9	a, b, c, d, e, f, h, i
3.1 Reasons for considering dropping out	5/7	a, b, e, f, h
*Concerns about the timeframe and future*	2/9	a, h
*Ambivalence about treatment*	4/9	b, e, f, h
*Stigma*	1/9	b
*External barriers*	3/9	e, f, h
3.2 Factors contributing to retention	4/9	b, c, d, e, h, i
*Seeing progress*	2/9	b, e,
*Encouragement from family and friends*	3/9	d, e, i
*Therapist support*	5/9	b, c, e, h, i
*Commitment*	1/9	e
*Wanting to believe in treatment*	3/9	b, e, i
3.3 Reasons for dropout	3/9	f, h, i
*External barriers*	3/9	f, h, i
*Therapy related difficulties*	3/9	f, h, i
*Therapeutic alliance issues*	1/9	f
*Lack of social support*	1/9	i
4. Perceived changes post-treatment	9/9	a, b, c, d, e, f, g, h, i
4.1 Perceived symptom changes	6/9	a, b, c, e, f, h,
*Reduced symptoms*	6/9	a, b, c, e, f, h
*Symptom exacerbation*	3/9	e, f, h
*Persistent symptoms*	4/9	b, c, e, h
4.2 Changed beliefs	8/9	a, b, c, d, e, g, h, i
*Changed beliefs about trauma*	6/9	a, b, d, e, g, h
*Changed beliefs about self*	7/9	a, b, c, e, g, h, i
*Changed beliefs about the future*	8/9	a, b, c, d, e, g, h, i
4.3 Better functioning	5/9	a, c, e, f, i

Frequency represents how many of the studies were represented in each domain, category, and sub-category. Studies represented in each category were coded by a letter a-i: Shearing et al. [53*] = a; Vincent et al. [54*] = b; Lowe and Murray [55*] = c; Murray et al. [56*] = d; Hundt et al. [57*] = e; Hundt et al. [58*] = f, Boterhoven de Haan et al. [59*] = g; Doran et al. [60*] = h; Eastwood et al. [38*] = i

#### Overcoming ambivalence towards TFT

Overcoming ambivalence towards TFT was identified as a major theme for all participants. This domain was further divided into three categories: Concerns about engaging in TFT, Desperation and hope facilitated engagement and The therapist’s role.

##### ***Concerns about engaging in TFT: fear and scepticism***

Participants described experiencing ambivalence about engaging in TFT. More specifically, participants expressed fear, anxiety, and avoidance. “I’d been avoiding it for ages and ages and ages, […]. And it wasn’t until I’d spoken, I’d thought it through, that I realised that I was scared of things, it was just kind of instinctive reaction of like horror, not wanting to go there” [53*] and “I am absolutely afraid I will get depressed again. In fact, I expect it” [60*].

##### ***Desperation and hope facilitated engagement***

Most of the studies described motivating factors that facilitated initiating TFT. Being desperate and hitting rock bottom was identified as a central factor for encountering the anticipated distress. Conversely, one study [53*] also identified being in a good place as essential for choosing to engage in TFT. Many highlighted the importance of having positive attitudes towards TFT. Three sub-groups emerged. One group reported feelings of hope, determination, commitment and being intrinsically motivated. A second group described emotional doubt but intellectual confidence in the effectiveness of therapy, which was promoted through the understanding of the treatment rationale and therapy process. The last group experienced scepticism and belief simultaneously.

##### ***The therapist’s role***

Most of the studies highlighted the therapists’ role in helping the patients overcome anticipatory anxiety and engage in trauma-focused interventions. Many studies emphasised the importance of the collaborative relationship, for example feeling that the therapist “facilitated” rather than “directed” the patients [55*] and experiencing being both pushed and respected [54*]. Furthermore, trusting the therapist was considered a significant factor in both empowering patients to manage complicated feelings [53*], expressing concerns about treatment [60*] and gaining a sense of self-efficacy [54*]. Participants identified several valuable characteristics of the therapist that facilitated trust: transparency regarding the content [38*], flexibility within therapy sessions [55*], attentive listening [54*], and empathy, understanding and non-judgement [58*].

#### Experience of treatment elements

All the studies described patients’ positive or negative experiences of specific elements of TFT, including perceptions of what aspects of therapy they valued or disliked. Despite some differences between the various types of interventions (TF-CBT, PE, CPT, reliving and site-visits as part of TF-CBT, ImRs and EMDR), most participants expressed they had changed their thoughts/beliefs (cognitive-behavioural element) and learned affective coping skills (affective modulation strategies). They also valued exposure and experiencing habituation (trauma-focused elements) and felt that homework generalised learning (homework assignments).

##### ***General experience of treatment***

Most of the studies included positive reports regarding cognitive-behavioural elements. Cognitive techniques such as questioning one’s thought processes and identifying cognitive biases were emphasised by participants as valuable in gaining a greater understanding of themselves, their triggers, symptoms, traumatic experiences, and were experienced as contributing to positive changes in thoughts/beliefs and better coping skills. In addition to cognitive strategies, affective modulation strategies and psychoeducation were both described as vital for change and helped the participants gain a sense of control. Regarding the length of therapy, there are mixed results. Some participants reported wanting more sessions, some were satisfied, and others were reported as finishing treatment early. See Table 3 for an overview of what articles report which of the reported themes.

##### ***Ambivalence and disagreement about trauma-focused elements***

Trauma-focused interventions received the most negative evaluation, but were also described as the most essential and critical parts of recovery. Since this category accounted for a large part of the analysis, it was separated into conflicting but complementary subcategories. The following subthemes show a disagreement in how the participants experienced trauma-focused interventions.

**Harder than expected versus unfounded fears**

Participants’ encounters with trauma-focused interventions varied and were categorised into three groups: (i) easier than anticipated, (ii) more challenging than expected, and (iii) difficult, as expected. Those individuals who perceived trauma-focused interventions as easier than anticipated described them as painful yet manageable, as their worst fears were proven unfounded. In the words of a participant, “Although it was very, very painful to relive it, I didn’t lose control, I didn’t scream, cry, or lash out” [53*]. A few studies reported experiences where treatment proved more challenging than expected, as expressed by a participant in Doran et al. [60*]: “I simply didn’t anticipate my reactions. I didn’t expect it to be this difficult”. In contrast, a majority experienced distress during the trauma processing, but recognized it as an expected and necessary part of the recovery process.

**Difficulty tolerating trauma work versus importance of hardship**

All studies consistently reported emotional distress among patients undergoing trauma-focused therapy (TFT), emphasising its demanding nature in addressing negative emotions and content. Common adverse reactions included initial symptom worsening, including nightmares, flashbacks, intrusive thoughts, and increased substance use [38*, 53*, 54*, 56*–58*].

Feeling overwhelmed during trauma-related exposures and re-experiencing the events was also frequently reported. Notably, one participant described the resurfacing of trauma-related emotions as “even more traumatic” than the original event, attributing this distress to an inability to disengage from the traumatic memories [38*]. Another participant conveyed a sense of dissociation during trauma work, stating, “… I was actually back when it was, all the stuff [the trauma] was happening. So I was like actually, I was scared and I’d leave, like obviously we’d stop because [the therapist] could see that I was not in the room” [38*].

Nonetheless, despite the distress experienced, discussing the trauma was described as “cathartic” and deemed essential [54*]. A majority of participants credited therapeutic progress to trauma work, emphasizing that the short-term pain was outweighed by the eventual benefits. One participant continued therapy even after relapsing into substance use during trauma narration, regarding it as a necessary part of their healing journey [58*]. However, it is important to note that not all participants shared this perspective, and dropout from treatment was often linked to an inability to manage the emotional distress associated with trauma work, which will be discussed separately.

**Life-consuming versus habituation**

Several participants reported that trauma work consumed every facet of their lives. This immersion manifested itself as persistent rumination and enduring low mood in the intervals between therapy sessions, which had adverse effects on their social, occupational, and academic aspects of life. For instance, one participant articulated their experience, stating, “I was very dark and depressed… constantly thinking about it, bringing up suppressed memories…” [38*]. Nonetheless, a prevalent pattern surfaced where participants progressively found the trauma-work more manageable over time, experiencing habituation and improved coping. They likened it to desensitisation, making it easier to discuss and express their experiences.

**Piecing together traumatic experiences versus wanting less repetition**

Participants generally had positive concluding remarks on trauma processing. Repeatedly discussing the “original event” facilitated memory reconstruction, changed beliefs [57*], memory vividness [56*], and “moving forward” [59*]. However, some wanted to address multiple events, patients who dropped out cited feeling overwhelmed by the repetitive sessions [60*], and some viewed discussing trauma as “dwelling on the past” and potentially hindering recovery [38*].

##### *Experience with homework*

Most participants found homework beneficial for practice and generalised learning [55*]. Positive experiences with homework served as positive feedback that the therapy was working [57*]. Challenges included finding the homework assignments confusing or having problems completing it due to other commitments [57*, 60*].

#### Motivation for dropout or retention

The third domain that emerged from the analysis was “Motivation for dropout or retention”. Although a minority of participants dropped out, many participants described wanting to drop out at some point. The domain was divided into the categories “Reasons for considering dropping out”, “Factors contributing to retention”, and “Reasons for dropout”.

##### ***Reasons for considering dropping out***

The primary reason for considering ending therapy was unresolved ambivalence about treatment, primarily rooted in doubts regarding therapy effectiveness, difficulty tolerating treatment, and avoidance behaviours, as indicated by one participant: “I thought about quitting because I was avoiding writing about my traumatic experience” [57*]. Stigma and cultural stereotypes surrounding seeking mental health prevailed and were a central theme for participants’ ambivalence about treatment. Concerns about treatment duration, especially its brevity, added to ambivalence, as participants questioned the adequacy of the allocated time. Uncertainty about the future, notably among asylum seekers awaiting decisions on their asylum claim, also led to doubts about the worth of continuing therapy. Furthermore, participants cited practical barriers, including transportation challenges and family responsibilities, as hindrances to therapy.

##### ***Factors contributing to retention***

Despite considering discontinuing treatment, most participants chose to continue therapy. Support from family and friends was mentioned by many as a primary reason for retention. For those lacking social support, the therapeutic relationship held great importance and openly discussing concerns with the therapist reinforced commitment. Additionally, perceived early progress, symptom improvement, relief and shifts in perspectives, inspired the patients’ persistence. Lastly, dedication to the therapy process, oneself, and the therapist was recognized as crucial during challenging phases, with some participants attributing their decision to continue to their “commitment” [57*].

##### ***Reasons for dropout***

Participants dropped out of therapy for four main reasons: external barriers, therapy-related difficulties, therapeutic alliance issues and lack of social support.

External barriers such as therapists lacking dedicated offices, scheduling conflicts, and limited health plan coverage, often led to disengagement. Therapy-related difficulties mirrored the concerns of those considering dropout. Many quit when they didn't see improvement or had doubts about the therapy approach, finding it too formal or preferring to learn coping skills. Some started therapies recommended by their therapists despite reservations [58*]. Interestingly, some dropped out despite believing in the benefits of trauma work due to an inner conflict between avoidance and recognizing the need for therapy [60*].

Participants expressed their willingness to consider returning to therapy if it were possible to develop a stronger therapeutic relationship, acquire enhanced coping skills, or resolve practical barriers [54*, 58*]. Importantly, those facing therapy-related challenges often cited issues with the therapist-patient relationship and a lack of social support [38*, 58*].

#### Perceived changes post-treatment

Participants reported noteworthy positive changes: reduction in symptoms, changed beliefs about the trauma, themselves and the future and better functioning. A few reported negative outcomes, which will be discussed within these categories.

##### ***Perceived symptom changes***

Positive symptom changes included reduced re-experiencing symptoms, improved sleep, mood, concentration, fewer nightmares, and less avoidance and hyperarousal. Most commonly, participants reported mood and functional improvements, a reduction in symptom intensity and frequency, while also acquiring coping skills to better manage residual symptoms. Yet, some saw no improvements, and a few felt worse, experiencing symptom exacerbation, nightmares, and substance abuse relapses [60*].

##### ***Changed beliefs***

Participants experienced profound shifts in their beliefs about trauma, themselves, and the future. These changes involved developing a new relationship with traumatic memories, reinterpreting the traumatic experiences, and gaining insights into their impact on life challenges. The changes extended to participants regaining a sense of agency, and experiencing bolstered confidence in handling trauma and reduced self-blame, exemplified by a participant recognizing they were allowed to be angry about what was done to them [53*]. This transformation indicated a profound shift in self-identity [38*, 57*].

Notably, asylum-seekers differed in their responses. They expressed feelings of weakness and illness due to their therapy needs, contrary to the more common experience of relief from shame and guilt. Accepting new perspectives was challenging for some asylum-seekers, leading to negative changes in self-understanding and beliefs about others [54*].

Despite these variations, most participants adopted a positive outlook on life following therapy. They expressed renewed hope for a meaningful future and a preference for life over death, representing a significant shift in their perspective.

##### ***Better functioning***

Symptom improvements, increased hope, and enhanced symptom management had a positive effect on various aspects of participants’ lives. They reported improved social relationships, occupational functioning, and a rekindled interest in previously enjoyed activities. For instance, one participant mentioned increased participation in family events [57*]. Another credited therapy with helping them open up about their experiences, which allowed for a sense of connection and social support [38*].

### Representativeness of findings

The representativeness of the findings in the review is presented in Table 3. Representativeness was assessed as (a) how many studies were represented in each category and sub-category, and (b) how many categories were identified in each study. Assessing the representativeness of each study in the findings was conducted by calculating the total amount of categories each study identified.

### Critical appraisal

The quality of the included studies was high and moderate, with six studies rated as high quality and three studies rated as moderate quality according to CASP. All studies gave a clear statement of the research aims, and qualitative research was justified and considered to be the suitable methodology for addressing the research goal. Recruitment bias was widespread throughout all the studies. For instance, two of the studies did not provide sufficient information about how the participants were selected [56*, 57*], and one study used self-selecting recruitment but did not discuss the implications of this method [53*]. Despite the presence of recruitment bias, most authors discussed their recruitment strategy (e.g., recruiting participants from one treatment facility or choosing participants who were thought to benefit from the treatment), which demonstrated systematic appraisal of study limitations. All the studies described and justified the data collection procedures. Five studies did not discuss the relationship between researcher and participants. This was mostly not considered to create a high risk of bias, as different individuals conducted the therapy, data collection and analysis. However, in two studies, participants might have been reluctant to disclose negative therapy experiences due to the nature of the data collection [54*, 55*]. Three studies did not provide sufficient details to assess whether ethical standards were maintained [53*, 55*, 56*]. All studies described data analysis rigorously. All except one study [56*] offered a comprehensive statement of findings. See Additional file 3 for a detailed description of the quality assessments.

## Discussion

The purpose of the present review was to understand and summarise patients’ experiences and dilemmas in trauma-focused therapy. The synthesis across patients with different trauma and six different types of TFT resulted in a common pattern, which provides insight into common experiences likely to occur throughout TFT. Comparing the experiences reported by those who considered dropping out but chose to stay and those who did drop out may help to illuminate, which aspects of TFT that are experienced as too challenging for patients [21, 27].

### Trajectories of treatment experiences

Different trajectories of how patients experienced the process of TFT emerged. Patients’ concerns about starting therapy, described in this review, concur with the common characteristics of PTSD, including avoidance and negative beliefs about the self, future, and trauma [18]. At the beginning of therapy, most participants experienced scepticism and fear of talking about the trauma. Despite the commonly held belief in both patients and therapists that the patients’ should be in “a good place in life” in order to engage in TFT, the most prevalently reported patient-experience in the study was that desperation (i.e., “hitting rock bottom”) was important for deciding to engage in TFT in the first place. Thus, apparently a combination of desperation and hope might compel patients to begin treatment.

Next, patients’ experiences with trauma-focused interventions were mixed. Importantly, most considered dropping out at some point, due to the hardship of trauma-focused interventions and doubts about its efficacy. This finding has important clinical implications, because while most TFT manuals prescribe motivational strategies before the initiation of trauma-focused exposure, to the best of our knowledge, none deal explicitly with retention strategies once exposure has begun (NET (15); CT-PTSD (14); CPT (10); PE (11)). Thus, therapists are currently left to devise their own retention strategies to the best of their ability.

Despite the high distress levels involved, difficulty tolerating treatment and overcoming the discomfort was found to be essential for recovery. Furthermore, overall the patient experiences did not support patients’ and therapists’ concerns that TFT might be ineffective or harmful. To the contrary, some described TFT to be effective, despite dropping out of therapy.

### Factors associated with retention

Factors associated with retention included buy-in to treatment, symptom improvement, fewer external barriers, social support, and a strong therapeutic relationship. Buy-in to treatment was crucial, often achieved through psychoeducation and therapist support.

The results indicated that buy-in to treatment can be divided into a rational, emotional and desperate buy-in. A rational buy-in was accomplished through thorough psychoeducation. An emotional buy-in was described as based on a trust in the therapists, facilitated by feeling both respected and pushed. When respected, patients were allowed time to understand the treatment rationale and take breaks, due to emotional limits. The experience of being pushed in a constructive way encompassed being exposed to trauma-interventions even when they did not believe they could tolerate it. The last group of clients, characterised by what we have termed desperate buy-in, preferred starting trauma intervention as soon as possible to limit the risk of them backing out. Despite not necessarily understanding the treatment approach, early trauma interventions allowed them to experience early symptom improvement. This exemplifies the complexities of trauma treatment and the all-important balancing of providing psychoeducation, ensuring “buy-in to treatment”, and early introduction of trauma-focused interventions. Hence, if we are to get better at preventing drop-out from TFT, designing explicit manualized strategies, which help therapists to recognize and address the above patterns, might be a way forward.

Patients perceived certain aspects of TFT as too inflexible, including the treatment content and process, consistent with concerns raised by therapists [21]. However, participants experiencing both being pushed and respected reported positive sentiments about its flexibility. Thus, although flexible sessions that adapt to individual needs while staying true to treatment goals may be beneficial, trusting the therapist might result in a more positive attitude towards the treatment manual. Overall, in order to improve retention, the expectations of treatment services regarding the pace and length of TFTs need to be flexible enough to allow for adaptations and good enough working alliances between therapists and patients, as well as good enough working conditions for those providing the treatments.

Social issues such as external stressors, logistical barriers and unstable living conditions often interfered with treatment. However, those reporting external barriers who simultaneously had a strong therapeutic relationship or good social support were more inclined to stay in therapy. Moreover, participants reporting both external barriers and lack of social support specifically emphasised the therapist’s support as a central factor in their decision to stay in therapy. For those experiencing alliance issues, discussing concerns about the treatment with their therapist often facilitated retention. This is in line with prior research that points to the significance of acknowledging factors related to the therapeutic alliance (i.e., the therapeutic relationship, empathy, support, and shared goals) as potential mediators of symptom change [61]. Finally, an important dynamic is demonstrated in the trajectory from the initial need to be in a place desperate enough to consider starting TFT, to the finding that those same social issues may become a hindrance in later phases of treatment. A clinical implication of this sequence might be that we should not wait for patients’ lives to become more “stable” before initiating TFT as has been suggested to reduce patients’ fear [62], but rather provide effective social counselling in parallel with TFT to alleviate the worst social stressors and improve retention during the exposure phase.

The findings shared similarities with the qualitative review of EMDR experiences among patients identified as benefiting from EMDR [36]. Similarities included the significance of a trusting therapeutic relationship to create a sense of safety and alleviate doubts and scepticism about therapy. Additionally, a common theme that emerged included experiencing a broader transformation through therapy, leading to an enhanced quality of life and changed beliefs about oneself.

### Treatment rationale of trauma-focused therapies

Patients’ experiences both aligned with and challenged the assumptions about common mechanisms promoting change in TFT through the targeting of emotion, cognition and avoidance, i.e., psychoeducation, exposure, memory processing and habituation [17].

Psychoeducation not only seemed to increase participants’ commitment to treatment but also positively influenced most patients’ self-perceptions and how they viewed their symptoms. These shifts in how they appraised their traumatic experiences were mainly attributed to cognitive techniques, while exposure interventions played a crucial role in memory modification and discrimination. Although a minority of patients did not experience habituation to their trauma memories or symptom improvement despite completing treatment, many still reported significant improvements representing an increase in quality of life [63], including better symptom management, increased sense of control, improved social relationships, and enhanced work and daily functioning.

While the positive outcomes might be attributed to or moderated by non-specific therapeutic factors, such as the therapeutic relationship [64], patients attributed the changes to the process of addressing their trauma. The key elements that patients valued in therapy included experiencing catharsis and relief through discussing their trauma, transforming their beliefs about trauma, the world, and themselves, and having a trustworthy and genuine therapist to facilitate these changes. In accordance with the theoretical framework proposed by Ehlers and Clark [18], the results indicate that from a patient perspective, trauma-focused work was essential for regaining trauma memory, achieving a new perspective on the trauma, and managing their symptoms.

### Diverse experiences within subgroups

The findings of the study indicate that Trauma-Focused Therapy (TFT) was experienced as beneficial for veterans and asylum seekers; however, the results suggest that these groups exhibit distinct and somewhat less favourable responses to TFT when compared to other populations. Notably, despite Clinical Practice Guidelines recommending TFT as the primary treatment for PTSD, recent research has uncovered inconsistent findings with regard to its superiority, particularly in the context of military-related PTSD [65–68].

These variations in treatment outcomes may stem from various external, cultural, or trauma-specific factors and the results underscore the imperative of cultural sensitivity when working with trauma survivors [69, 70]. Both veteran and asylum seeker groups encountered significant challenges related to stigma, shame, and guilt. Stigma could exacerbate negative appraisals of trauma and self-perception, subsequently impeding help-seeking behaviour. This, in turn, could contribute to the persistence and severity of symptoms, as noted in prior research [71]. Moreover, limited help-seeking behaviour among these groups may lead to reduced social support, a factor which has been identified as crucial for maintaining engagement and retention in treatment.

Asylum seekers’ difficulties in acknowledging past traumas and altering their beliefs about themselves and the world pose a crucial challenge in the context of most TFTs. Among the other study samples, gaining a new perception of the trauma memory appeared as essential for improving symptom management, aligning with existing studies demonstrating that alterations in appraisals mediate the change of PTSD symptoms [72]. This observed subgroup difference might reflect significant cultural variations in the perception of psychiatric diagnoses and trauma experiences. However, it might be that current threats moderate this relationship. Notably, the fear of repatriation emerged as a significant barrier hindering engagement for asylum seekers. This aligns with theoretical models suggesting that the perception of current threats sustains PTSD symptoms [18]. Despite evidence supporting the effectiveness of TFT for individuals at high risk of re-exposure [25], the results suggest that the coexistence of cultural stigma and current threats may increase the likelihood of treatment dropout among these patient populations. More qualitative studies of refugee-patients’ experiences with TFT are needed to better understand the obstacles to effective implementation of TFT in this group.

### Clinical implications

Creating a safe therapeutic environment is crucial to enhance the effectiveness and tolerability of Trauma-Focused Therapy (TFT). Addressing and working through ambivalence plays a central role in building trust in treatment and influencing patient engagement and retention. Trust encompasses both a rational understanding of the treatment rationale and an emotional reliance on the therapeutic process and therapist.

Therapists should prioritise building trust, considering the client's unique subjective and cultural perspectives. It is particularly important to inform the patient about the treatment principles, to present a clear rationale for the exposure component that acknowledges that revisiting the trauma memories may seem counterintuitive, and to provide realistic expectations for both positive and negative experiences throughout therapy. It is important to anticipate and openly discuss common reactions like fear, ambivalence, and symptom exacerbation, reassuring clients that these are typical and not indicative of treatment failure. Finally, anticipating patients’ ambivalence during treatment and manualizing retention strategies in TFTs might be a way of improving their effectiveness.

For individuals hesitant to engage in treatment due to apprehension, the awareness of shared experiences or the recognition that encountering challenges like relapse and symptom worsening has been reported as worthwhile by others may be reassuring. Follow-up sessions aimed at discussing re-engagement are recommended, as most dropouts express a willingness to retry TFT.

Some participants, particularly refugees and veterans, express a desire to move on from traumatic events, which contrasts with TFT's core element of revisiting trauma for processing. Therapists should explain this counterintuitive approach and address client scepticism through education and preparation, both in sessions and through written or online materials. Patients across the studies emphasised the importance of understanding the treatment rationale, suggesting a need for adaptations like translated materials or culturally relevant language for minority groups [73].

Clinicians should address the potential stigma and increased sense of guilt associated with psychiatric diagnosis for multiple marginalised groups. Challenges including shame, isolation and cultural stereotypes suggest that a group-oriented approach focusing on these aspects as part of psychoeducation, could be beneficial. Recognizing that cultural context shapes trauma experiences, the results suggest that the treatment should address the perception of current stressors alongside past trauma, rather than exclusively concentrating on a single traumatic event. The findings suggest that patients with many social stressors might benefit from more short-term intensive treatment [74, 75], or/and treatments that focus on alleviating social stressors in parallel with provision of TFT.

This review informs psychoeducation by shedding light on TFT experiences and elucidating common treatment patterns. Referring to how other patients respond can assist clinicians and patients in developing trust in TFT. Recognizing that ambivalence and fear can deter both patients and therapists, this review may mitigate treatment dropouts and enhance willingness to engage in TFT.

### Strengths and limitations

A strength of this review is the pre-defined inclusion and exclusion criteria. The wide range of search terms and the inclusion of hand-searched articles enhanced the likelihood to detect a large proportion of existing papers relating to patients’ experiences of TFT. The studies represented both positive and negative experiences, providing a nuanced picture of patients’ experiences. The studies included a diversity of sub-groups (veterans, asylum seekers, youths, and patients with various index traumas), allowing for a more comprehensive understanding of typical and distinct experiences seen across these groups, e.g., difficulties with completing TFT. The studies reviewed were conducted in the US, UK, Australia, Germany and Netherlands, with a wide range of ethnicities represented.

The included studies covered a variety of therapeutic methods (imaginal reliving, site-visits, PE, CPT, Adapted Testimony, ImRe and EMDR). Furthermore, the quality of this systematic review was strengthened through credibility checks. The authors amongst themselves encompassed a broad range of therapeutic experiences and preferences, including both TFT-practitioners and non-TFT practitioners. Lastly, the representativeness of the findings in this systematic review was assessed [47].

Nonetheless, certain limitations affect the generalizability of the findings. Participants with a positive experience could have been more inclined to participate in the research. Also, the use of purposive sampling might have contributed to sampling bias, resulting in overly optimistic reports about TFT experiences. For example, certain studies included patients who completed at least eight sessions of therapy [57*], who found the treatment to be a positive experience [55*], or who were believed to benefit from exposure interventions [56*, 60*]. Furthermore, interviews conducted in the setting where the participants received therapy might have impacted to what degree the participants were willing to disclose negative experiences [54*]. Finally, the included studies do not represent the perspectives of patients who have refused to start in TFT. It is likely that studies investigating the expectations and concerns of patients who are not willing to enter into a therapy including an exposure component would have further added to the understanding of patient perspectives on TFT.

Despite the possibility that some studies contributed to disproportionately positive results, it was also evident in the studies that most participants shared both negative and positive experiences. Furthermore, certain of the included studies actively reduced the possibility of overgeneralizing positive findings in the review by focusing on those who had dropped out of TFT [38*, 58*, 60*]. Even when bearing in mind the impact of the potential methodological biases, the themes reflecting positive and negative experiences were similar across different types of study populations. Moreover, dropouts from all three studies reported positive feelings about TFT, and many were willing to try TFT again.

Despite the inclusion of several therapeutic interventions, most studies examined therapies related to TF-CBT and no studies examined the experience of narrative exposure therapy. Approximately half of the sample and 70% of the dropouts were veterans. The exclusion criteria could have biased the reported results, as non-English language studies and studies not published in peer-reviewed publications were excluded. The exclusion of grey literature could have contributed to a publication bias of the reported experiences. Furthermore, the reporting of the results (a few, some, many, several and most) might represent a bias. Due to a lack of accurate descriptions in some of the studies, it was impossible to provide a precise number of participants reporting different experiences. Two authors selected relevant “quotations” from the original studies, and although means were taken to stay close to the language of the original studies when describing the results, both the original researchers of the primary studies and the authors of the current review might have overlooked some information. Nevertheless, by conducting rigorous and systematic analysis, the findings are likely to offer a comprehensive and in-depth picture of patients’ experiences.

### Future research

First, it is evident that there is a lack of studies involving TFT experiences of multiply marginalised groups. Future research should prioritise investigating how these specific populations experience TFTs. Given that most existing studies focus on TF-CBT, it is crucial to expand our understanding by exploring how patients experience Narrative Exposure Therapy (NET). It would be relevant to focus such studies on refugees or asylum seekers, as this group did not experience the common trajectory of altered beliefs about themselves, the trauma and the world, and because NET was developed to treat trauma within this population and has demonstrated effectiveness [76]. The literature search also identified a lack of studies investigating therapists’ experience with conducting TFTs. Further research should investigate both patients’ and therapists’ experiences, making it possible to compare their experiences and to what extent they align.

## Conclusion

Overall, participants reported high levels of distress and re-emergence of symptoms during trauma work. Still, despite negative experiences, most patients were grateful and perceived the hardship as essential for improvement. At the beginning of therapy, most participants experienced scepticism and fear of talking about the trauma. Most participants expressed a reduction in ambivalence throughout therapy due to experiencing symptom improvement, understanding the treatment rationale, and trusting the therapist’s empathy and expertise. Some expressed persistent ambivalence about the effectiveness of the treatment as an essential factor for dropping out. Also, it seems that it is vital for participants to be informed about the treatment principles and the rationale for the exposure component of the therapy. Clinicians should emphasise that therapy will be challenging and that symptom exacerbations may occur during trauma exposure, but that these experiences do not imply that treatment does not work. Instead, experiencing difficulties with the treatment and overcoming these could be essential for recovery. Most participants experienced significant improvements in symptoms and quality of life. Realistic expectations of symptom improvements post-therapy should be emphasised, as many still experienced some symptoms post-treatment. However, therapy helped them gain coping skills, a sense of control, agency and a better outlook on life. The results also emphasised that participants’ appreciated hearing about other people’s experiences, as it helped them overcome their ambivalence about staying in therapy.

### Supplementary Information

**Supplementary Materials 1. **

**Supplementary Materials 2. **

**Supplementary Materials 3. **

## Data Availability

All data generated or analysed during this study are included in this published article and its supplementary information files.

## Abbreviations

CPT: Cognitive processing therapy
CT-PTSD: Cognitive therapy for post-traumatic stress disorder
EMDR: Eye movement desensitisation and reprocessing
NET: Narrative exposure therapy
PE: Prolonged exposure
PTSD: Post-traumatic stress disorder
TF-CBT: Trauma-focused cognitive behaviour therapy
TFT: Trauma-focused therapy
