# Supplementary material for: How is trauma-focused therapy experienced by adults with PTSD? A systematic review of qualitative studies
Source: BMC Psychol. 2024 Mar 9;12:135. doi: 10.1186/s40359-024-01588-x (PMC10924413; doi:10.1186/s40359-024-01588-x)
Supplement: Supplementary file 1 — Supplementary Materials 1. [file 40359_2024_1588_MOESM1_ESM.docx]

Additional file A: Search Strategy

| Database (Date) | Searches | Results |
| --- | --- | --- |
| PubMed (30.11.21) | # 1: "post traumatic stress disorder*"[All Fields] OR "posttraumatic stress disorder*"[All Fields] OR "PTSD"[All Fields] OR "stress disorders, post-traumatic"[MeSH Terms] |  |
|  | #2: “cognitive processing therap*” OR “prolonged exposure*” OR OR "Eye Movement Desensitization and Reprocessing" OR “Narrative Exposure therapy” OR “Brief Eclectic psychotherapy” OR “Written Narrative Exposure” OR TF-CBT OR (“Trauma-focused” AND (“therapy” OR “psychotherapy” OR “cognitive behavior therap*” OR “cognitive behaviour therap*” OR “cognitive behavioural therap*” OR “cognitive behavioral therap*” OR “behavior therap*” OR “behaviour therap*” OR “behavioural therap*” OR “behavioral therap*” OR “behavior psychotherap*” OR “behaviour psychotherap*” OR “behavioral psychotherap*” OR “behavioral psychotherap*” OR “cognitive therap*” OR “cognitive psychotherap*” OR “cognition therap*”)) |  |
|  | #3: “self report*” OR “patient report*” OR “client report*” OR experienc* OR view* OR perspective* OR perce* OR opinion* OR understand* OR reflect* OR reaction* OR thought* OR Standpoint* OR “Patient receptivit*” OR “Client receptivit*” OR satisfaction* OR “client record*” OR “patient record*” OR attitude* OR feel* OR Belie* OR know* OR thought* OR standpoint* |  |
|  | #4: questionnaire* or survey* or interview* or focus group* or “case stud*” or observ* or qualitative* or “thematic analy*” or content analy* or ethnog* or phenomenol* or emic or etic or hermeneutic* or “heuristic*” or semiotics or “field study*” or “lived experience*” or “narrative analy*” or “grounded theor*” or “multi-method*” or “mixed-method*” or triangula* or “formative evalua*” or “process evalua*” |  |
|  | #5: qualitati* or “mixed-meth*” or “mixed meth*” or “multi-meth*” or “multi meth*” |  |
|  | #6: #1 AND #2 AND (#3 OR #4) AND #5 |  |
|  | #7: #6 AND filters: Journal Article, Humans, English, Middle Aged: 45-64 years, Young Adult: 19-24 years, Adult: 19-44 years, Middle Aged + Aged: 45+ years, Aged: 65+ years, 80 and over: 80+ years | 45 |
| PsycINFO (30.11.21) | #1: «Posttraumatic stress disorder*» OR «Post-traumatic stress disorder*” OR “Post traumatic stress disorder*” OR PTSD |  |
|  | #2: “cognitive proces* therap*” OR “Prolonged exposure*” OR TF-CBT OR "Eye Movement Desensitization and Reprocessing" OR “Narrative Exposure therapy” OR “Brief Eclectic psychotherapy” OR “Written Narrative Exposure” OR (“Trauma-focused” AND (“therapy” OR “psychotherapy” OR “cognitive behavio#r* therap*” OR “cognitive therap*” OR “behavio#r* therap*” OR “behavio#r psychotherapy*” OR “cognit* therap*” OR “cognit* psychotherap*”)) |  |
|  | #3: «Self-report*» OR «Patient* report*» OR «Client* report*» OR «Experience*» OR view* OR perspective* OR perce* OR opinion* OR understand* OR reflect* OR reaction* OR thought* OR Standpoint* OR “Patient* receptivit*” OR “Client* receptivit*” OR satisfaction* OR “client record*” OR “patient* record*” OR attitude* OR feel* OR Belie* OR know* OR thought* OR standpoint* |  |
|  | #4 questionnaire* or survey* or interview* or focus group* or “case stud*” or observ* or qualitative* or “thematic analy*” or content analy* or ethnog* or phenomenol* or emic or etic or hermeneutic* or “heuristic*” or semiotics or “field study*” or “lived experience*” or “narrative analy*” or “grounded theor*” or “multi-method*” or “mixed-method*” or triangula* or “formative evalua*” or “process evalua*” |  |
|  | #5 qualitati* or “mixed-meth*” or “mixed meth*” or “multi-meth*” or “multi meth*” |  |
|  | #6: #1 AND #2 AND (#3 OR #4) AND #5  Limits: Peer Reviewed; Publication Type: Peer Reviewed Journal; Language: English; Age Groups: Adulthood (18 yrs & older), Young Adulthood (18-29 yrs), Thirties (30-39 yrs), Middle Age (40-64 yrs), Aged (65 yrs & older), Very Old (85 yrs & older); Population Group: Human | 74 |
| PTSDPubs (30.11.21) | #1: «Posttraumatic stress disorder*» OR «Post-traumatic stress disorder*” OR “Post traumatic stress disorder*” OR PTSD |  |
|  | #2: “cognitive processing therap*” or “prolonged exposure*” or TF-CBT OR "Eye Movement Desensitization and Reprocessing" OR “Narrative Exposure therapy” OR “Brief Eclectic psychotherapy” OR “Written Narrative Exposure” OR (“Trauma-focused” AND (“therapy” OR “psychotherapy” OR “cognitive behavior therap*” or “cognitive behaviour therap*” or “cognitive behavioural therap*” or “cognitive behavioral therap*” or “behavior therap*” or “behaviour therap*” or “behavioural therap*” or “behavioral therap*” or “behavior psychotherap*” or “behaviour psychotherap*” or “behavioral psychotherap*” or “behavioral psychotherap*” or “cognitive therap*” or “cognitive psychotherap*” or “cognition therap*”)) |  |
|  | #3: “self report*” OR “patient report*” OR “client report*” OR experienc* OR view* OR perspective* OR perce* OR opinion* OR understand* OR reflect* OR reaction* OR thought* OR Standpoint* OR “Patient receptivit*” OR “Client receptivit*” OR satisfaction* OR “client record*” OR “patient record*” OR attitude* OR feel* OR Belie* OR know* OR thought* OR standpoint* |  |
|  | #4: questionnaire* or survey* or interview* or focus group* or “case stud*” or observ* or qualitative* or “thematic analy*” or content analy* or ethnog* or phenomenol* or emic or etic or hermeneutic* or “heuristic*” or semiotics or “field study*” or “lived experience*” or “narrative analy*” or “grounded theor*” or “multi-method*” or “mixed-method*” or triangula* or “formative evalua*” or “process evalua*” |  |
|  | #5: qualitati* or “mixed-meth*” or “mixed meth*” or “multi-meth*” or “multi meth*” |  |
|  | #6: #1 AND #2 AND (#3 OR #4) AND #5 AND limits: peer reviewed, English, adults, peer reviewed; source type: scholarly journal; document type: journal article; Language: English; exclude duplicate documents; NOT (Literature Review AND Review) | 44 |
| Total results |  | 163 |
