# Supplementary material for: How is trauma-focused therapy experienced by adults with PTSD? A systematic review of qualitative studies
Source: BMC Psychol. 2024 Mar 9;12:135. doi: 10.1186/s40359-024-01588-x (PMC10924413; doi:10.1186/s40359-024-01588-x)
Supplement: Supplementary file 2 — Supplementary Materials 2. [file 40359_2024_1588_MOESM2_ESM.docx]

Additional file B: Reasons for exclusion

| Article | Reason for exclusion | Description |
| --- | --- | --- |
| Tong et al. (49) | Wrong population | The study describes the inclusion criterion as having current trauma symptoms, operationalized as meeting the full criteria for PTSD as assessed using Clinician Administered PTSD Scale (CAPS), or having clinical-level dissociation, or exposure to trauma (i.e., the participants did not necessarily meet the criteria for PTSD). |
| Hundt et al. (28) | Wrong objective | The study investigated participants’ perceived barriers and facilitators to begin TFT. Did not fulfil the inclusion criterion of exploring patients’ experiences of undergoing treatment. |
| König et al. (50) | Wrong objective | Compared patients’ written responses of what they thought was helpful and not helpful in one session of dialogical exposure therapy or cognitive processing therapy. The study compared the importance of treatment elements without a description of how these treatment elements were experienced |
| Sherrill et al. (51) | Wrong objective | Investigated patients’ perceived benefits and drawbacks of intensifying the PE protocol. The study did not include qualitative information about how the patients’ experienced the treatment elements or trauma-focused interventions. Instead, participants were asked to describe the benefits and drawbacks of daily therapy and imagine the difference between weekly and daily treatment |
| Wise & Marich (52) | Wrong objective | Investigated how individuals with co-occurring PTSD and addictive disorders perceived EMDR addiction-specific protocols as a mechanism of change. The authors did not explore the patients’ experiences and perceptions of treatment but asked how the treatment had impacted their understanding of the interrelatedness of the interrelatedness of the addictive symptoms and traumatic memories and how these symptoms had changed. |
