# Supplementary material for: How is trauma-focused therapy experienced by adults with PTSD? A systematic review of qualitative studies
Source: BMC Psychol. 2024 Mar 9;12:135. doi: 10.1186/s40359-024-01588-x (PMC10924413; doi:10.1186/s40359-024-01588-x)
Supplement: Supplementary file 3 — Supplementary Materials 3. [file 40359_2024_1588_MOESM3_ESM.docx]

Additional file C: Scores for each study based on CASP

|  | Shearing et al. (53) | Vincent et al. (56) | Lowe & Murray (55) | Murray at el. (58) | Hundt et al. (59) | Hundt et al. (57) | Boterhoven de Han et al. (60) | Doran et al. (54) | Eastwood et al. (38) |
| --- | --- | --- | --- | --- | --- | --- | --- | --- | --- |
| 1. | Yes | Yes | Yes | Yes | Yes | Yes | Yes | Yes | Yes |
| 2. | Yes | Yes | Yes | Yes | Yes | Yes | Yes | Yes | Yes |
| 3. | Yes | Yes | Yes | Yes | Yes | Yes | Yes | Yes | Yes |
| 4. | No | Yes | Yes | No | No | Yes | Yes | Yes | Yes |
| 5. | Yes | Yes | Yes | Yes | Yes | Yes | Yes | Yes | Yes |
| 6. | No | Yes | Partially | No | Partially | Partially | Yes | No | Yes |
| 7. | No | Yes | Partially | No | Yes | Yes | Yes | Yes | Yes |
| 8. | Yes | Yes | Yes | Yes | Yes | Yes | Yes | Yes | Yes |
| 9. | Yes | Yes | Yes | Yes | Yes | Yes | Yes | Yes | Yes |
| Total | 6 | 9 | 8 | 6 | 7,5 | 8,5 | 9 | 8 | 9 |

Retrieved from: Critical Appraisal Skills Programme (39). 1. Was there a clear statement of the aims of the research? 2. Is a qualitative methodology appropriate? 3. Was the research design appropriate to address the aims of the research? 4. Was the recruitment strategy appropriate to the aims of the research? 5. Was the data collected in a way that addressed the research issue? 6. Has the relationship between researcher and participants been adequately considered? 7. Have ethical issues been taken into consideration? 8. Was the data analysis sufficiently rigorous? 9. Is there a clear statement of findings? 10. How valuable is the research?

Yes: 1

Partially: 0.5

No: 0

Scores 0 - 3: low quality

Scores 4 - 7: moderate quality

Scores 8 - 9: high quality

*Comments and potential biases for each study based on CASP*

***Shearing et al****.* (53)

*4. Was the recruitment strategy appropriate to the aims of the research?*

Recruitment was done through self-selecting (information sheets) which might have increased the likelihood of a sample of participants who had benefitted from therapy. Information about those who did not partake was not available.

*6.* *Has the relationship between researcher and participants been adequately considered?*

First author completed the interviews. However, no discussion is presented about the relationship between interviewers and participants (this might not necessarily be relevant or create a bias).

*7. Have ethical issues been taken into consideration?*

Ethical issues have not been taken into consideration.

***Vincent et al.*** (56)

Was rated “yes” for all questions because the biases were described in limitations, but still important to consider.

*4. Was the recruitment strategy appropriate to the aims of the research?*

The sample was homogenous in three aspects: all had experienced traumatic events, received CBT involving TF-CBT, and were seeking asylum in the UK. However, due to the small pool of potential participants, there was diversity in the sample regarding age, gender, country of origin, traumas experienced and stage in treatment.

Furthermore, clinicians did not approach clients who had disengaged from therapy. TF-CBT may have been offered to clients less preoccupied with threats of repatriation, and with more stable social circumstances, and therapists may have invited clients to participate who had seemingly benefited from therapy. Participants may have differed from non-English speaking clients, who would have encountered interpreters in therapy.

*6. Has the relationship between researcher and participants been adequately considered?*

Biases the researchers describe: “Participants may have been reluctant to disclose negative experiences of therapy due to being interviewed in settings where they had received therapy, or because of high appreciation and regard for clinicians. However, most participants reported difficulties experienced in therapy.”

*10. How valuable is the research?*

Valuable, however with some biases and restraints for generalizability of the findings, and differences or potential influences of the results due to the population. The authors highlight these limitations, but they should be taken into consideration when evaluating the findings of the review.

***Lowe & Murray*** (55)

*4. Was the recruitment strategy appropriate to the aims of the research?*

Only one was a specialist in PTSD outpatient treatment service.

*6. Has the relationship between researcher and participants been adequately considered?*

The interview was conducted by the therapist while the analysis was conducted by a clinical psychologist and a peer group of experienced qualitative researchers. The article does not examine the therapists’ role, e.g., potential bias in the interviews of their clients.

*7. Have ethical issues been taken into consideration?*

Ethical approval was obtained in accordance with the authors’ institutional research and governance procedures, however there were not sufficient details on how the research was explained to participants for the reader to assess whether ethical standards were maintained.

***Murray et al.*** (58)

*4. Was the recruitment strategy appropriate to the aims of the research?*

Study participants were a convenience sample, recruited across two adult outpatient services in London, a traumatic stress service and a specialist centre for anxiety disorders and trauma. The generalisability of the results is limited because the participants were a pre-selected group, based on the therapists’ evaluation of whether the exposure would be therapeutically beneficial.

*5. Was the data collected in a way that addressed the research issue?*

The participants received a questionnaire after a visit to the scene of the trauma, which was appropriate for the aims of the study - to explore whether participants found site-visits helpful. However, the questionnaire only included three free text items for qualitative analysis, which limited the article’s second aim of making a grounded theory of how site-visits were experienced. The questions were 1. What was experienced as helpful and unhelpful; 2. Suggestions for what may have made the site visit more helpful; 3. If you did find the photo helpful, what was helpful about it?

*6. Has the relationship between researcher and participants been adequately considered?*

The researchers have not examined their role, potential bias and influence during data collection and sample recruitment. The participants were given a questionnaire and might have been reluctant to disclose negative experiences of therapy.

*7. Have ethical issues been taken into consideration?*

There are not sufficient details of how the research was explained to participants. Issues around informed consent and confidentiality are not provided.

***Hundt et al.*** (59)

*4. Was the recruitment strategy appropriate to the aims of the research?*

There was no discussion about recruitment and why some people chose not to take part. Of the 40 letters sent, 23 patients (57%) agreed to participate, 13 (33%) declined participation, and 4 (10%) could not be contacted. Also, as described in limitations, “although this study examined veterans receiving treatment from a variety of therapists, only veterans from one VA medical site, rather than community providers, were recruited”.

*5. Was the data collected in a way that addressed the research issue?*

The authors describe the use of qualitative interview guide and audio recording, the process of modification of topic guide, however, they don’t specify if it was a semi-structured interview.

*6.* *Has the relationship between researcher and participants been adequately considered?*

The authors did not provide sufficient information about the researchers’ or interviewer’s role, potential bias, and influence during data collection. However, interviews were conducted by the first author and two other members of this research team with qualitative interview experience, supervised by a medical sociologist trained in qualitative methods (J.A.).

***Hundt et al.*** (57)

*6.* *Has the relationship between researcher and participants been adequately considered?*

Interviews were conducted by the first author and another member of this research team with qualitative training. Does not discuss relationships and bias (however, this might not be important).

***Boterhoven de Han et al.*** (60)

*4. Was the recruitment strategy appropriate to the aims of the research?*

The recruitment strategy was appropriate, however, there was no discussion about why some people chose not to take part. In the limitation section, the authors discussed that the participants may not generalize to other Ch-PTSD patients because they had been through the screening process and initial assessment including agreeing to 12 sessions of trauma processing before initiating treatment.

***Doran et al.*** (54)

*4. Was the recruitment strategy appropriate to the aims of the research?*

“Participants were offered the option to participate in a psychotherapy process and outcome study in the clinic after independently electing to engage in an Evidence based practice (EBP) for PTSD*.* This might create a bias, as the participants themselves chose EBP.

*5. Was the data collected in a way that addressed the research issue?*

Interviews were not tape-recorded but transcribed, which opens for error of misunderstanding (the authors describe this limitation themselves). The interviews were structured, but open-ended.

*6.* *Has the relationship between researcher and participants been adequately considered?*

Does not say who completed the interviews.

*10. How valuable is the research?*

The research was based on a small sample size and was not representative as it was restricted to a homogenous sample (white male veterans) who engaged in one PTSD clinic in a New England CA and chose to participate in EBP. Responses might not generalise to those who chose not to participate. However, the research provides valuable qualitative information on the perceptions and experiences of a small sample of veterans.
